# Supplementary material for: CRISPR/Cas9-Mediated Multiplexed Genome Editing in Aspergillus oryzae
Source: J Fungi (Basel). 2023 Jan 13;9(1):109. doi: 10.3390/jof9010109 (PMC9864741; doi:10.3390/jof9010109)
Supplement: Supplementary file 1 [file jof-09-00109-s001.zip › jof-2093893-supplementary.pdf]

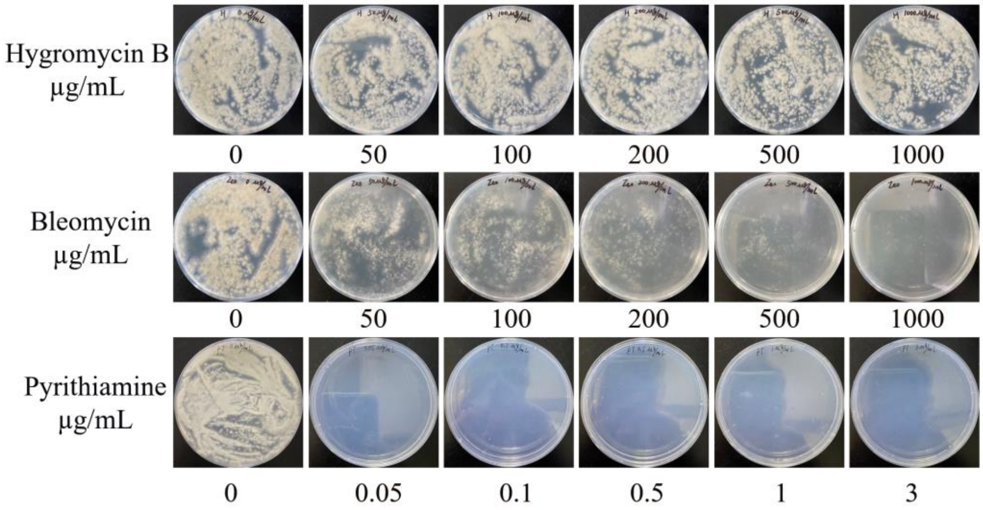

Figure S1. The sensitivity of *A. oryzae* to commonly used antibiotics. *A. oryzae* RIB40 is not sensitive to hygromycin. Compared with bleomycin, pyrithiamine inhibited the growth of *A. oryzae* more obviously.

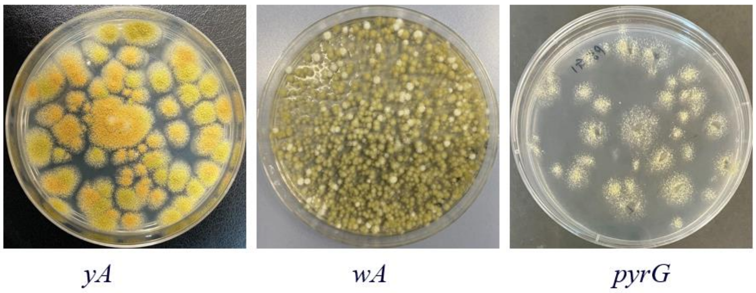

Figure S2. Transformation plates for random editing efficiency statistics. Mutants of the *yA* gene encoding conidial laccase form yellow conidia, whereas *wA* mutants form white conidia due to the lack of the polyketide synthase required for conidia coloration.

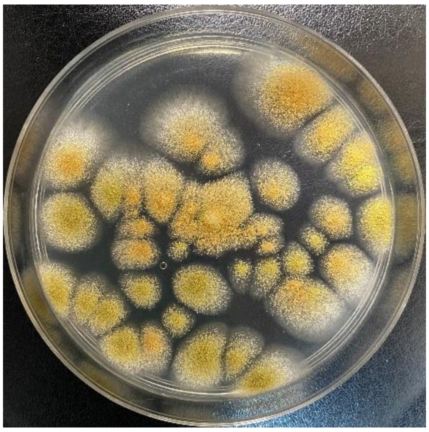

Figure S3. Transformation plates for integrated expression of lipase. The plasmid pTR-C9sgR-yA-amyB was transformed into *A. oryzae* together with the donor DNA PamyB-lipase-TamyB.

Table S1 Primers used in this study

Primer	Sequence (5’-3’)
Edit-assemble-F	CAATTTCACACAGGAAACAGCTATGACCATGATTACGCCAC ATAGCTGTTTCCGCTGAGG
Edit-assemble-R	GGTACCCGGGGATCCTCTAGAGTCGACCTGCAGGCATGCA GACGTATGCGTCCCTGGGAG
Protospacer-yA-F	TTCTCACTAAGTTTTAGAGCTAGAAATAGCAAG
Protospacer-yA-R	TCATTTGGCGTGCATCATCCGTGAATCG
Protospacer-wA-F	TAAGCCGGTGGTTTTAGAGCTAGAAATAGCAAG
Protospacer-wA-R	TGGCTCTTAGTGCATCATCCGTGAATCG
Protospacer-pyrG-F	CGGCTCCGAGGTTTTAGAGCTAGAAATAGCAAG
Protospacer-pyrG-R	TAGGGGAAGTTGCATCATCCGTGAATCG
Protospacer-Ku70-F	AAGCGTTGTGGTTTTAGAGCTAGAAATAGCAAG
Protospacer-Ku70-R	GCAATCTGCGTGCATCATCCGTGAATCG
Protospacer-amylase -F	CTTGAGATGAGTTTTAGAGCTAGAAATAGCAAG
Protospacer-amylase-R	TGCAGTGCCGTGCATCATCCGTGAATCG
Protospacer-agdA-F	ACCCACCATAGTTTTAGAGCTAGAAATAGCAAG
Protospacer-agdA-R	AGACGCTCTCTGCATCATCCGTGAATCG
Locus-amyB-F	GGCTTGTATTGTTGCGACGC
Locus-amyC-F	AGAGACGGATGGTTGCGTAG
Locus-TLL-R	ACCATCATGACCTCTACAACCTGAAC

Table S2 CRISPR/Cas9 mediated single-gene editing in *A. oryzae*. The length of the homology arm of Donor DNA is 1000 bp. $e = Rm * Ec + (1-Rm) * En$ for *yA* and *wA*. $e = Rp * Ep$ for *pyrG*.

Repair pathway	Target gene	Target and PAM sequence	Indels	editing efficiency/%			
				Rm/*Rp*	*Ec*/*Ep*	*En*	*e*
NHEJ	*yA*	CGCCAAATGATTCTCACTAATGG	1-1402 bp	37	100	0	37
HR			2186 bp	17.5	100	0	17.5
NHEJ	*wA*	CTAAGAGCCATAAGCCGGTGAGG	1-1325 bp	37.6	100	0	37.6
HR			1500 bp	35.3	100	0	35.3
NHEJ	*pyrG*	ACTTCCCCTACGGCTCCGAGAGG	1-514 bp	36.7	100	-	36.7
HR			899 bp	21.9	100	-	21.9

Table S3 CRISPR/Cas9 mediated dual gene editing in *A. oryzae*. *e=e1 * e2*.

	Target gene	Target and PAM sequence	Indels (the latter)	*e1*/% (yA)	*e2*/% (in yellow)	*e*/%
NHEJ	*yA+agdA*	CGCCAAATGATTCTCA CTAATGG (*yA*)<br>GAGAGCGTCTACCCAC CATACGG (*agdA*)	1-176 bp	19.8	100	19.8
HR			1725 bp	19.6	100	19.6
NHEJ	*yA+amyB*	CGCCAAATGATTCTCA CTAATGG (*yA*)<br>CGGCACTGCAGATGAC TTGAAGG (*amyB*)	1-107 bp	18.5	100	18.5
HR			1981 bp	18.9	100	18.9
NHEJ	*yA+Ku70*	CGCCAAATGATTCTCA CTAATGG (*yA*)<br>CGCAGATTGCAAGCGT TGTGAGG (*Ku70*)	1-11 bp	6.5	95	6.3
HR			715 bp	17	100	17
